# An Image Encryption Algorithm Based on Time-Delay and Random Insertion

**DOI:** 10.3390/e20120974

**Published:** 2018-12-15

**Authors:** Xiaoling Huang, Guodong Ye

**Affiliations:** Faculty of Mathematics and Computer Science, Guangdong Ocean University, Zhanjiang 524088, China

**Keywords:** image encryption, time-delay, random insertion, information entropy, chaotic map

## Abstract

An image encryption algorithm is presented in this paper based on a chaotic map. Different from traditional methods based on the permutation-diffusion structure, the keystream here depends on both secret keys and the pre-processed image. In particular, in the permutation stage, a middle parameter is designed to revise the outputs of the chaotic map, yielding a temporal delay phenomena. Then, diffusion operation is applied after a group of random numbers is inserted into the permuted image. Therefore, the gray distribution can be changed and is different from that of the plain-image. This insertion acts as a one-time pad. Moreover, the keystream for the diffusion operation is designed to be influenced by secret keys assigned in the permutation stage. As a result, the two stages are mixed together to strengthen entirety. Experimental tests also suggest that our algorithm, permutation– insertion–diffusion (PID), performs better when expecting secure communications for images.

## 1. Introduction

With fast development of computer and network technologies, digital information (multimedia) modalities (such as images, video, and audio) have been widely adopted for daily communication. Among these, image analysis is a most direct and simple way to learn and understand the natural world. Images are increasingly transformed over networks every day, according to the Google analysis. Images and applications utilizing image processing are used in many fields, such as medicine, education, and aerospace, to name a few. However, illegal attackers may visit, read, or intercept our transmitted information.

Cryptology can be utilized to develop methods for secure transmission of images. However, images are different from text files, and have many unique characteristics, such as bulk data capacity, high redundancy, and strong inter-pixel correlation. As a result, traditional encryption algorithms such as DES, AES, and IDEA are not suitable for secure encoding of images. Development of algorithms for effective image encryption remains an important priority in the fields of computer science and communications. Recently, chaos-based image encryption schemes have received considerable attention; these methods allow for hiding image-related information accounting for the desirable properties [1,2] of extreme sensitivity to initial conditions, ergodicity, and pseudo-randomness of chaos systems (maps). As early as 1998, Fridrich [3] proposed an image encryption method that used a two-dimensional chaotic map. In what follows, many encryption algorithms have been designed, which fully or partially utilize the Fridrich structure (i.e., permutation–diffusion). For example, a bit-level image encryption algorithm [4] was proposed based on piecewise linear chaotic maps, in which a diffusion strategy was introduced followed by a permutation of bits for each value. Quantum chaotic map [5] with a diffusion-permutation architecture-based image encryption algorithm has been presented. Norouzi et al. designed diffusion-only image encryption schemes [6,7]. The test results show high sensitivity and high complexity. The behavior of quantum walks was proved [8] to be chaotic, and a permutation-based image encryption algorithm has been proposed. It calculates the sum of the plain-image and uses the resulting value to diffuse the image’s pixels. Furthermore, to enhance the sensitivity of the encryption method, a quantum hash function is taken to act as a hash function for the privacy amplification process [9]. Exclusive OR (XOR) as a diffusion operation and shuffling as a permutation are then applied to the plain-image and yield a cipher-image with a new encryption structure. A unique and more distinctive encryption algorithm is proposed based on the complexity of a highly nonlinear S box in Flesnelet domain [10]. DNA-based image encryption methods [11,12,13] and other similar architectures [14,15,16,17,18,19,20,21,22] have also been presented as encryption techniques to ensure communication of images.

However, some schemes have been found to be insecure. For example, Li [23] evaluated a class of permutation-only encryption algorithms. Using a known(chosen)-plaintext attack, the plain-image could be recovered if the encryption algorithm [24] was used. Furthermore, it was shown how permutation-only image encryption schemes can be broken with little computation complexity [25,26]. Eslami and Bakhshandeh [27] designed a new image encryption to promote the plain-text sensitivity and to enhance the diffusion performance. However, the keystream used in that diffusion was not related to the plain-image. As a result, Akhavan et al [28] re-evaluated the security and broke it successfully using a chosen plain-text attack [27]. Other cryptanalysis methods [29,30,31,32] have been proposed as well.

To solve the above security problem and to enhance the connection between the plain-image and the keystream, a novel chaotic image encryption scheme, named permutation–insertion–diffusion (PID), is proposed in this paper. A middle parameter is designed to revise the outputs of the chaotic map, acting like a time-delay phenomena. To enhance the security of the Fridrich structure, especially the shortcoming of unchanged gray values before the diffusion operation [33,34], a group of random numbers are inserted in the pre-encrypted image to rewrite the gray distribution followed by the diffusion encryption. As a result, the proposed algorithm can be seen as a one-time pad. The rest of this paper is organized as follows. The proposed cryptosystem is described after an introduction of a chaotic map. Then, some experimental results are shown by using our method. After that, security analyses are evaluated to explain the better performance of our scheme. Finally, conclusions are drawn followed by a discussion.

## 2. The Proposed Cryptosystem

**Figure 1:**
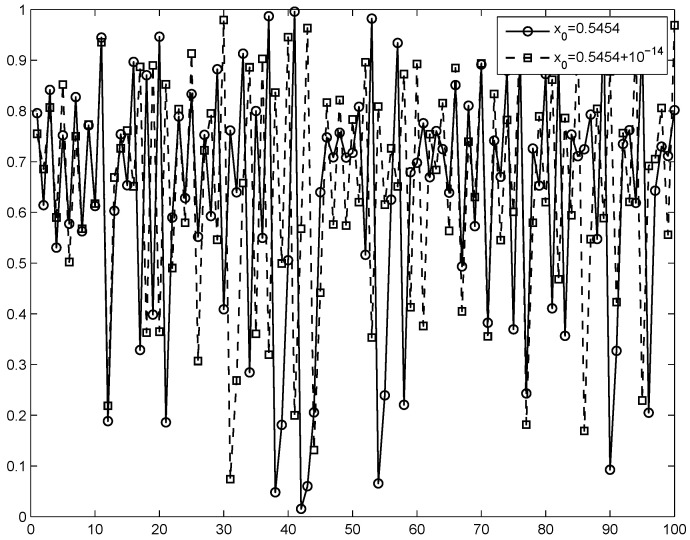
Chaotic dynamics in the 2D-SLMM map.

A two-dimensional (2D) chaotic map, called a 2D Sine Logistic modulation map (2D-SLMM), was studied in [34]. The map is defined by

(1) $$\left\{\begin{array}{c} x_{i+1}=u\left(\sin\left(\pi y_{i}\right)+v\right)x_{i}\left(1-x_{i}\right),\\ y_{i+1}=u\left(\sin\left(\pi x_{i+1}\right)+v\right)y_{i}\left(1-y_{i}\right),i=0,1,2\cdots , \end{array}\right.$$

where u∈[0,1], v∈[0,3]. To enhance the nonlinearity and the randomness, parameter *v* is set to modulate the output of the Logistic map. When we let v be close to 3, the output pairs $\left(x_{i+1},y_{i+1}\right)$ of 2D-LASM distribute in the whole data range of the 2D phase plane. Thus, *v* is set to be 3 [34] in 2D-SLMM to display good chaotic performance. Figure 1 shows the chaotic orbit for the 2D-SLMM output. A detailed description of the map is provided in [34].

### 2.1. Image Cryptosystem

To deduce the strong correlation among adjacent pixels in the plain-image, pixel shuffling is considered as a first step. Let *P* be an m×n plain-image, and randomly set initial conditions u=0.9966, v=3.000, $x_{0}=0.4237$ and $y_{0}=0.1784$ in the permutation stage. Then, after a certain number of iterations, two sets $\left\{x_{i}\right\}$ and $\left\{y_{i}\right\}$ are obtained. To generate a keystream with a time-delay-like phenomenon, the following operations are performed:

(2) $$\left\{\begin{array}{c} s=1+\sum P_{i,j},\\ t=\lceil y_{i}\times 10^{14}+s\rceil \;\mathrm{mod}\;7+1,\\ {\bar{x}}_{i}=x_{i+t},\\ h_{i}=\left\lceil 3{\bar{x}}_{i}\times 10^{14}\right\rceil \;\mathrm{mod}\;n+1,i=1,2,\cdots ,\\ l_{j}=\left\lceil 5{\bar{x}}_{j}\times 10^{14}\right\rceil \;\mathrm{mod}\;m+1,j=1,2,\cdots , \end{array}\right.$$

where ⌈a⌉ corresponds to the floor operation on *a*, and *t* is a time-delay factor. As a result, we obtain $H=\{h_{1},h_{2},\cdots ,h_{m}\}$ and $L=\{l_{1},l_{2},\cdots ,l_{n}\}$ for circular permutations of row and column. Assume that the permuted image is *T* after permutation encryption.

If permutation-only operation is applied to a plain-image, then the gray distribution of the permuted image is the same as that of the plain-image. Moreover, encryption schemes in this family were found to be insecure. To enhance the security level of the proposed algorithm, random numbers are inserted into the image *T* before the first row with a random row *a* and the first column with a random column *b*. A new image *B* is obtained, with the dimensions of (m+1)×(n+1). As a result, the gray distribution of *B* is different from that of image *P*. Fortunately, the insertion function acts as a one-time pad owing to the random numbers being generated anew each time. For example, vector a={3,8,9,20} may become a={11,34,5,7} randomly with a four-dimension. Thus, the obtained cipher-images are different, even if the encryption is performed on the same plain-image in different communications.

To determine the relationship between the different pixels in the cipher-image, diffusion is further used to encrypt the permuted image *B*. Again, random initial conditions are set as u=0.9966, v=3.000, ${\hat{x}}_{0}=0.6028$ and ${\hat{y}}_{0}=0.1883$ in the diffusion stage, and the chaotic map is iterated. Then, a chaotic matrix *M* with the same size as *B* is obtained after a certain number of iterations. To revise the gray distribution, the following operation is performed on the matrix rows:

(3) $$\left\{\begin{array}{c} D=B+M\;\mathrm{mod}\;256,\\ \;r=\lceil \left(x_{0}+y_{0}\right)\times 10^{14}\rceil \;\mathrm{mod}\;7+1,\\ \;C_{i}=C_{i-1}+rM_{i}+D_{i}\;\mathrm{mod}\;256,i=1,2,\cdots ,m+1, \end{array}\right.$$

where $C_{i}$, $M_{i}$, and $D_{i}$ represent the row vectors of *C*, *M*, and *D*, respectively. $C_{0}$ is a constant vector. Finally, a cipher-image *C* is obtained. It is noted that, before the diffusion operation, a rewriting operation for the permuted image *B* should be performed, which overcomes the shortcoming of the Fridrich structure and enhances the encryption security. Considering a similar function in the case of columns, the above function by row is applied again, this time on the columns of image *C*, and the following cipher-image *E* is obtained:

(4) $$\left\{\begin{array}{c} F=C+M\;\mathrm{mod}\;256,\\ E_{j}=E_{j-1}+rM_{j}+F_{j}\;\mathrm{mod}\;256,j=1,2,\cdots ,n+1. \end{array}\right.$$

### 2.2. Encryption Steps

As described above, the proposed encryption scheme can be summarized in the following steps, with the symmetric PID structure:

Step 1. Read the plain-image as *P* and obtain its size m×n.
Step 2. Compute the sum *s* over the plain-image.
Step 3. Generate the two sets *H* and *L* by simulating a time-delay phenomena.
Step 4. Apply circular permutation to both rows and columns, and obtain *T*.
Step 5. Insert random numbers into the permuted image *T* and obtain *B* by simulating a one-time pad.
Step 6. Iterate the chaotic map again and obtain matrix *M*.
Step 7. Apply the diffusion operation to revise the gray distribution, on both row and column dimensions.
Step 8. Obtain the cipher-image *E*.

### 2.3. Decryption

Owing to the symmetric cryptosystem nature of our method, image decryption can be performed by applying the same steps in reverse, starting from the ciphered image and ending up with the plain image. Using correct keys, the diffusion operation is firstly applied, followed by the permutation operations in the reverse order.

## 3. Experimental Results

**Figure 2:**
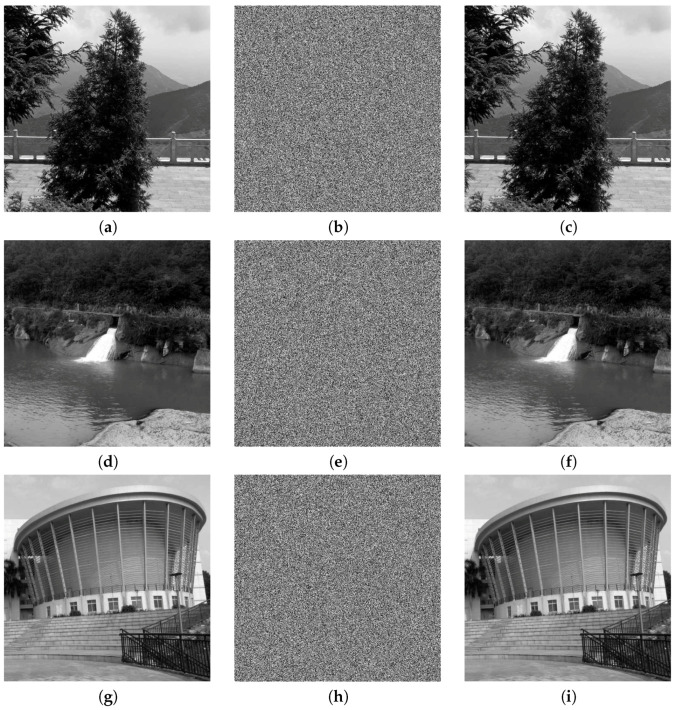
Experimental tests: (**a**) plain-image of Tree; (**b**) cipher-image of Tree; (**c**) decrypted image of Tree; (**d**) plain-image of Lake; (**e**) cipher-image of Lake; (**f**) decrypted image of Lake; (**g**) plain-image of Building; (**h**) cipher-image of Building; (**i**) decrypted image of Building.

Three images were randomly chosen, tests were performed using our proposed method, and the results are reported in this section. The test was implemented in Matlab 2011b running on Windows 7 (Notebook with Intel(R) Core(TM) i3-2350, 2.30 GHz CPU). To increase the security, the PID process was applied twice to each image. Then, the former 100 iteration results for the chaotic map were deleted to avoid harmful effects. Figure 2 shows the plain-images, corresponding cipher-images, and their decrypted results. It is clear that these cipher-images contain no useful image-related information, compared with their corresponding plain-images.

## 4. Security Analyses

### 4.1. Key Space Analysis

The key space corresponds to the space of all combinations of keys that can be used in a certain encryption scheme. Here, there are four keys, i.e., $x_{0}$, $y_{0}$, ${\hat{x}}_{0}$, ${\hat{y}}_{0}$, not including the parameters *u*, *v*. The key space becomes as large as $10^{56}\approx 2^{186}$ if the precision is set to $10^{-14}$. As a result, it is difficult to conduct a successful brute-force.

### 4.2. Histogram Analysis

**Figure 3:**
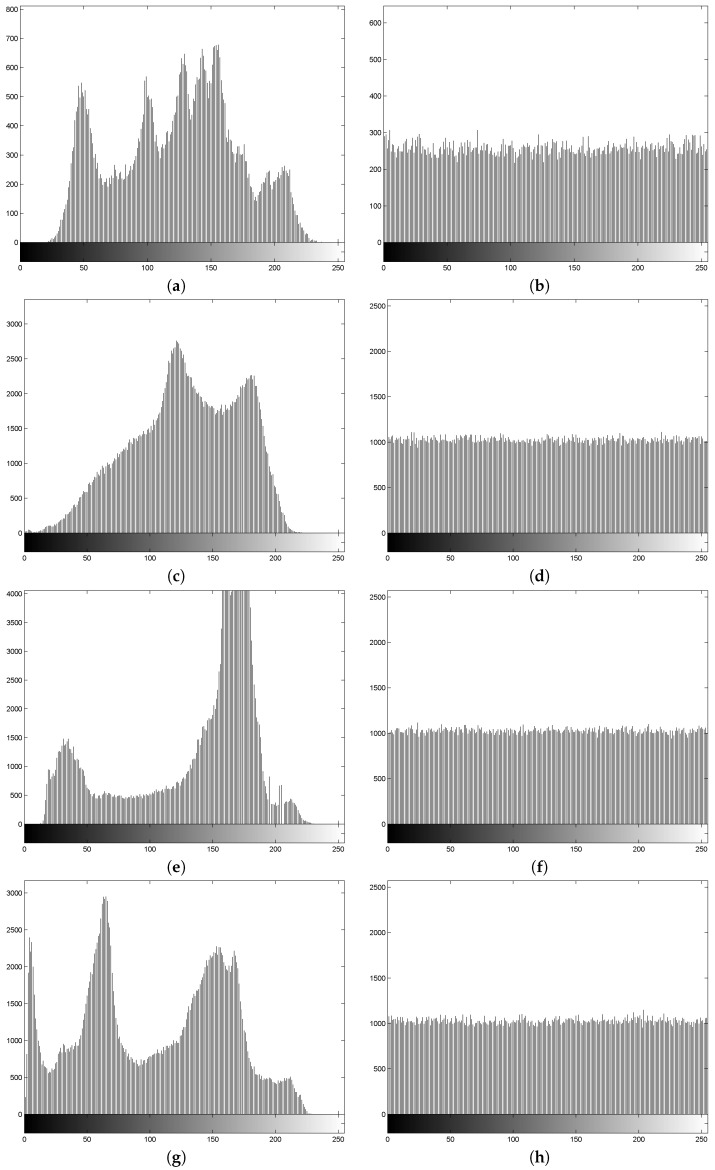
Histograms of: (**a**) the plain-image of Lena; (**b**) the cipher-image of Lena; (**c**) the plain-image of Baboon; (**d**) the cipher-image of Baboon; (**e**) the plain-image of Boat; (**f**) the cipher-image of Boat; (**g**) the plain-image of Peppers; (**h**) the cipher-image of Peppers.

To reduce the chance of attack and to efficiently hide the information of a plain-image, the histogram of the corresponding cipher-image should be uniform and significantly distinct from that of the plain-image. Figure 3 shows the histograms for the images of Lena, Baboon, Boat, and Peppers before and after using our encryption scheme. It is clear that the histograms of the encrypted images are flat. Thus, successful attacks are impossible.

### 4.3. Information Entropy Analysis

**Figure 4:**
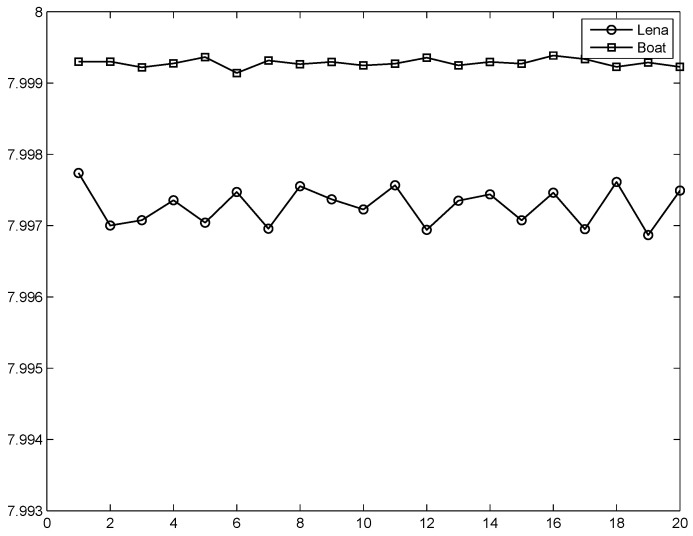
Information entropy at different times of encryption.

Information entropy [1] is an efficient measure of the randomness of an input image (message). This measure can be defined using the following equation:

(5) $$E\left(m\right)=\sum_{i=0}^{L-1}p\left(m_{i}\right)log_{2}\frac{1}{p(m_{i})},$$

where $L=2^{k}$ is the total number of states of the tested message (k=8 for a gray level image). Here, we tested four images, and the results are listed in Table 1 (using code “entropy” in Matlab). We conclude that the information entropy indicates that it is difficult to conduct a successful attack because the values of the information entropy for the cipher-images are close to a theoretical value of 8 [35,36]. The random numbers inserted into the image in each encryption, so the values of the information entropy would be changed very slightly each time. Figure 4 shows the results for encrypting Lena and Boat at different times.

### 4.4. Key Sensitivity Analysis

**Figure 5:**
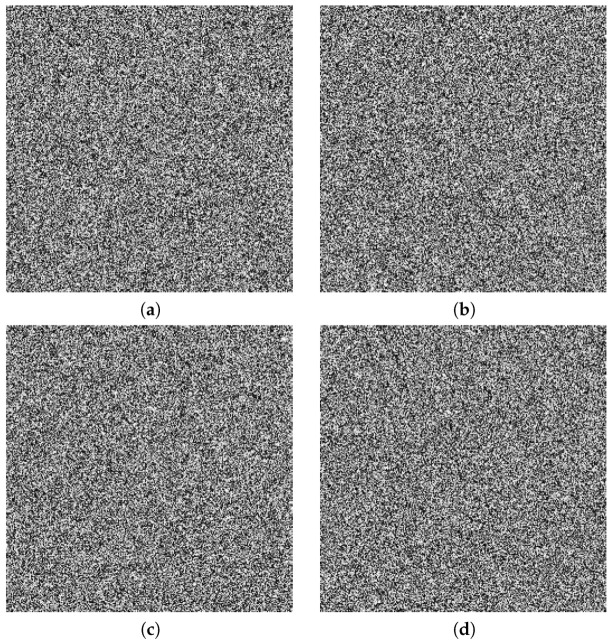
Key sensitivity tests for Lena: (**a**) decryption with $x_{0}+10^{-14}$; (**b**) decryption with $y_{0}+10^{-14}$; (**c**) decryption with ${\hat{x}}_{0}+10^{-14}$; (**d**) decryption with ${\hat{y}}_{0}+10^{-14}$.

A good image encryption algorithm should be very sensitive to all of the keys used. We tested our algorithm on the image of Lena, and the results are listed in Figure 5. Figure 5a–d shows the incorrect decryption from the cipher-image with a small change (i.e., $10^{-14}$) added in keys $x_{0}$, $y_{0}$, ${\hat{x}}_{0}$, and ${\hat{y}}_{0}$, respectively. Therefore, the proposed image encryption algorithm possesses a high key sensitivity and can frustrate brute-force attackers.

### 4.5. Differential Analysis

To test the sensitivity of the proposed encryption method to a small change, even one bit, in the plain-image, we used two common measures [37,38], the number of pixel change rate (NPCR) and the unified average changing intensity (UACI). The measures are defined as follows:

(6) $$NPCR=\frac{\sum_{i,j}D\left(i,j\right)}{m\times n}\times 100\%,$$

(7) $$UACI=\frac{1}{m\times n}\sum_{i,j}\frac{|C^{\prime }\left(i,j\right)-C\left(i,j\right)|}{255}\times 100\%,$$

(8) $$D\left(i,j\right)=\left\{\begin{array}{c} 0,\;\mathrm{if}\;C^{\prime }\left(i,j\right)=C\left(i,j\right),\\ 1,\;\mathrm{otherwise}, \end{array}\right.$$

where $C^{\prime }$ and *C* are two cipher-images corresponding to the same plain-images differing only in one bit. The results of this test are listed in Table 2 for a change in the value of the (100,89) position. The results in Table 2 show that our method has high sensitivity to changes in the plain-images because the values are nearly ideal [39].

### 4.6. Run Test for Randomness

The run test mainly examines whether the probability of an event is random. In Matlab software, ”runstest” performs a run test on a given sequence *X*. This is a test of the hypothesis that the values in *X* come in a random order. If the sequence is random, then the test result is 0, or the result is 1. By using our algorithm, the test results are listed in Table 3. Therefore, the outputs show good statistical randomness.

### 4.7. Comparisons

To make a comparison, information entropy was taken to measure the randomness of different plain-images and their corresponding cipher-images. Here, a color image of Lena was selected for comparison with some methods [1,38,40]. The results are given in Table 4 for tests on cipher-images. Obviously, the information entropy values are close to the ideal value of 8 for our proposed scheme. Furthermore, computational complexity is an important metric for measuring the efficiency of the designed algorithm. Table 5 compares the proposed algorithm with some recent references, for different sizes. Considering key size, information entropy, and running speed, Table 6 displays the comparisons for some of other methods [41,42,43,44,45], where information entropy is tested for a cipher-image. Thus, our method can show good performance to satisfy a real-time communication.

## 5. Discussion and Conclusions

In this paper, an image encryption scheme was proposed that utilizes a chaotic map. This paper makes four significant and novel contributions: (1) The keystream used in the permutation stage is affected by the plain-image, (2) a time-delay phenomenon is simulated and constructed for choosing chaotic outputs, (3) a group of random numbers are inserted into the permuted image before diffusion, and (4) the keystream used in the diffusion stage is affected by the keys assigned in the permutation stage. According to the results of some tests and security analyses, the proposed image encryption scheme exhibits a good performance and is suitable for application in secure communications.

An image encryption algorithm based on time-delay and random insertion with a PID structure was investigated in this paper. With the help of chaotic map as key generator and its inherent properties, time-delay was simulated by outputs of chaotic map. Then, random numbers are inserted before diffusion operation to remedy the shortcoming of Fridrich structure. Compared with previous works, the proposed image encryption algorithm has the following features:

(1) High sensitivity to keys and the plain-image.
(2) Time-delay phenomenon is simulated according to outputs of the chaotic map.
(3) One-time pad is designed by inserting random numbers before diffusion.
(4) The keystream used in the diffusion stage is affected by keys assigned in the permutation stage.
(5) Faster speed to implement the encryption.

## Figures and Tables

**Table 1:** Information entropy tests.

Test Images	Plain-Image	Cipher-Image
Lena	7.4532	7.9970
Boat	7.1238	7.9993
Peppers	7.5715	7.9992
Baboon	7.3579	7.9993

**Table 2:** Sensitivity tests.

Test Images	UACI	NPCR
Lena	33.3537	99.6109
Boat	33.4899	99.5900
Peppers	33.5186	99.6044
Baboon	33.5280	99.6136

UACI: unified average changing intensity; NPCR: number of pixel change rate.

**Table 3:** Run test for randomness.

Images	Lena	Peppers	Boat	Baboon
Results	0	0	0	0
Randomness	Pass	Pass	Pass	Pass

**Table 4:** Comparisons of information entropy.

Channels	R	G	B	Average
Ref. [1]	7.9903	7.9890	7.9893	7.9895
Ref. [38]	7.9871	7.9881	7.9878	7.9877
Ref. [40]	7.9278	7.9744	7.9705	7.9576
Ref. [46]	7.9969	7.9974	7.9970	7.9971
Ref. [47]	7.9895	7.9897	7.9893	7.9895
Ref. [48]	7.9968	7.9970	7.9972	7.9970
Ours	7.9977	7.9973	7.9975	7.9975

**Table 5:** Comparisons of speed performance.

Sizes	Ref. [46]	Ref. [47]	Ref. [48]	Ours
256×256	0.1641 s	0.0552 s	0.0671 s	0.0312 s
512×512	0.6630 s	0.2031 s	0.2293 s	0.1373 s

**Table 6:** Comparisons by gray Boat image.

Sizes	Key Size	Information Entropy	Running Speed	Software
[41]	$2^{298}$	7.9993	21.684 s	Matlab
[44]	$2^{128}$	7.9993	5.960 s	Matlab
ours	$2^{186}$	7.9992	0.137 s	Matlab
